# Overcoming barriers to implementing brief intervention: a knowledge transfer partnership

**DOI:** 10.1186/1940-0640-8-S1-A38

**Published:** 2013-09-04

**Authors:** Paul Jordan, Jonathan Shepherd, Simon Moore

**Affiliations:** 1 Violence and Society Research Group, Cardiff University, Cardiff, UK

## 

Cardiff University, the Welsh Government and Public Health Wales are working together to implement screening and brief interventions across a variety of health and community settings through a unique collaboration; a Knowledge Transfer Partnership (KTP). Previous research has uncovered both barriers and facilitators to implementation. This KTP seeks to build upon this work and translate the research into practice. The Have-a-Word social marketing initiative, the first of its kind, was launched in 2013 in Wales and seeks to specifically address the barriers to implementing SBI. To date over 2000 health and community professionals have received training^1^. This paper demonstrates how social marketing has been used to overcome barriers to implementing screening and brief interventions across a variety of health and community settings in Wales. The Have-a-Word campaign, currently being rolled out across Wales, seeks to manage the image of screening and brief interventions and encourage those who have received training to deliver brief interventions. Evaluation of the Welsh model shows that many health and community professionals who received training continue to view a formal screening tool such as FAST as a barrier to successfully implementing the SBI model in specific settings. Social marketing has helped overcome barriers to delivery (some nurses commented that they felt part of an SBI community which empowered them). A more pragmatic approach to screening is required since the formal screening tool was dissuading some professionals from delivering brief interventions at all. Social marketing campaigns can help manage the image of brief interventions however sustaining those who have received training remains a challenge and continued support and encouragement are needed.

^1^This includes maxillofacial and trauma clinic nurses, midwives, health visitors, dieticians, custody sergeants, youth workers and practice nurses.

